# Wages and employment security following a major disaster: A 17-year population-based longitudinal comparative study

**DOI:** 10.1371/journal.pone.0214208

**Published:** 2019-03-29

**Authors:** Peter G. van der Velden, Ruud J. A. Muffels, Roy Peijen, Mark W. G. Bosmans

**Affiliations:** 1 CentERdata, Tilburg, The Netherlands; 2 Tilburg University’s Network on Health and Labor (NETHLAB), Tilburg, The Netherlands; 3 Intervict, Tilburg University, Tilburg, The Netherlands; 4 TRANZO, Tilburg School of Social and Behavioral Sciences, Tilburg University, Tilburg, The Netherlands; 5 Department of Sociology, Tilburg School of Social and Behavioral Sciences, Tilburg University, The Netherlands; 6 Tilburg Institute of Governance (TIG), Tilburg University, The Netherlands; 7 NIVEL, Utrecht, The Netherlands; University of Zurich, SWITZERLAND

## Abstract

**Objectives:**

The effects of disasters on mental health are well documented, but very little is known about the short to long-term effects of human-made disasters on wage and employment security careers of the affected residents.

**Methods:**

Residents affected by a major fireworks disaster (May 13, 2000) in a Dutch residential area were all anonymously identified, based on postal codes of the affected area. To gain insight in these effects, data were derived from Statistics Netherlands that records all individual demographic, gross annual wages and employment security data of the entire Dutch population since 1999. A quasi-experimental matched control group design was used by constructing two pair-wise matched groups of non-affected residents of the city of Tilburg and the general Dutch population. Matching was based on nine demographic variables such as gender, age, education level and gross annual wage in 1999 (N^total^ = 12,648). The effects of the disaster on wage and employment security from 1999 to 2016 among the total group and among those with low wages in 1999, were assessed using fixed-effects panel regression analyses.

**Results:**

Affected residents had significant lower gross annual wages in the medium and long term than the non-affected groups from the Netherlands, but differences were (very) small. Compared to the Tilburg group the significant differences were trivial in the medium term. Among the low-wage groups, no relevant differences were found between affected and non-affected residents. With respect to employment security, no or trivial differences were found between the total group of affected and matched comparison groups. Among those with low wages in 1999, in 2001 and especially 2002 affected residents worked fewer weeks per year than non-affected from Tilburg. In 2002 the difference with the Tilburg group was above moderate.

**Conclusions:**

These results speak to the resilience of affected residents, given the mental health problems and PTSD-symptomatology they suffered from, as shown in previous research.

## Introduction

Each year and across the globe, residents become victim of human-made or technological disasters, such as terrorist attacks, airplane and large-scale industrial accidents. A large body of research has demonstrated that a variable minority of the affected residents develop a mental disorder such as posttraumatic stress disorder, generalized anxiety and major depression disorder [1–4]. However, the burden of a disaster on mental health is not restricted to mental disorders [2, 4]. Disaster studies have documented that many more suffer from PTSD-, depression- and other stress-related symptoms but do not meet the strict diagnostic criteria of mental disorders according to the Diagnostic and Statistical Manual (DSM, APA) or International Classification of Diseases (ICD, WHO). In general, the prevalence of event-related mental disorders and mental health problems declines as time passes by, but the rate of recovery differs between disasters [1–4].

To date, multiple factors have been identified that increase the risk for post-disaster mental health problems, varying from pre-event mental health problems [5] and lack of social cohesion [6] to acute job loss due to the event [7] and financial problems following the event [8], showing that they are multifactorial determined. Post-disaster mental disorders and mental health problems may negatively affect the way affected residents perform at work, as is the case with non-trauma related mental health problems [9–17]. Mental disorders and mental health problems are associated with for instance sickness leave, declining work performance, sustained work disabilities, perceived workload, low job satisfaction, and job loss that may subsequently impact adversely their gross annual wages and employment security [9–14]. The relationships may be bi-directional, such as that unemployment may lead to mental health problems [15]. In addition, unemployment or a badly paid job at the start of the career may impose scars on individuals’ future careers resulting into reduced employment probabilities and reduced wages [16, 17].

A fundamental or core question in this perspective is to what extent human-made disasters influence the development of the wage and employment security among the affected residents: do post-event annual wages and employment among affected residents differ from comparable non-affected residents in the short, medium and long term? Both wages and employment security are important resources in life according to the Conservation of Resources theory of Hobfoll [18] and loss of these resources may cause (additional) stress and negatively affect well-being. Unemployment is characterized by a process of loss of resources from all the categories suggested by Hobfoll, i.e. object resources (such as car and house), condition resources (such as employment and marriage), personal resources (such as skills and self-esteem), and energy resources (such as knowledge and money).

Previous studies on this topic among affected residents mainly focused on the short-term effects of natural disasters on employment growth and rates, such as hurricanes, tornados and floods, instead of man-made disasters [19]. In general, major natural disasters such as the Tangshan earthquake (1976), the Katrina disaster (2005) and the Haiti earthquake (2010), affected large areas including the (partial) destruction of local factories and other commercial and non-commercial business. For instance, following earthquakes that occurred in El Salvador in early 2001, it was estimated “*that 32*,*540 jobs were lost and another 9*,*200 jobs are at risk*, *implying approximately 23*.*4 million dollars in lost income in the period of six to 18 months required for establishments to be repaired page*”(see page 109 in [19]). In a study following a tornado (2000) and based on data from the US Bureau of Labor Statistics, Ewing and colleagues [20] found different patterns in employment growth between sectors. Some sectors showed no differences in pre- and post-tornado employment growth in the two years post-event (construction, finance, insurance, real estate, government, and transportation and public utilities), whereas other sectors (manufacturing, service, wholesale, and retail trade) showed falling levels of employment growth. Kosanovich [21] reported that, also based on aggregated data of the US Bureau of Labor Statistics, during the first two months following Hurricane Katrina payroll employment in Louisiana declined by almost 250,000, while it increased by more than 75,000 in the US in the same period. Again, large differences were found between sectors in the first two months. During the first ten months, post-event Louisiana's employment was still below pre-Katrina level (more than 1.9 million versus less than 1.8 million employed people). The study of Zissimopoulos and Karoly [22], based on individual-level data from the nationally representative monthly Current Population Survey (CPS) in the US, showed that 71% of the evacuees of Katrina participated in the labor force after the event. Among prime-age individuals from non-Katrina states and non-evacuees from Katrina states, these percentages were 79% and 77% respectively. The difference in employment security between non-returnees and returnees was 38% versus 70% over the 12-month period, indicating higher employment rates among returnees.

Relatively few studies were aimed at employment security and wages after man-made disasters. Rasco and North [23] assessed the employment and relationships with PTSD up to three years post-event among an aggregated sample of victims of five human-made and two natural disasters which took place before 1992. They concluded that long-standing employment disability in the three years post-event, was virtually nonexistent in their study samples. Lehman and Wadsworth [24] examined the long-lasting effects of exposure to the Chernobyl disaster on the health and labour market performance of the adult workforce using data of three surveys (2003, 2004, 2007) of the large Ukrainian Longitudinal Monitor Survey event, thus 17–21 years post-event. Results showed that residents who lived in the affected areas in 1986 were six to eight percentage points less likely to be in work than those living elsewhere at that time. Due to the absence of data on employment security in the first decade after the event, it is unknown whether the differences between both groups already existed before or in the first years of the post-event period. In contrast to many human-made or technological disasters this major disaster however, like major floodings, affected and destroyed a very large area.

To the best of our knowledge, to date no longitudinal disaster study is available that examined the gross annual wages and employment security in the short, medium and long term among residents affected by a human-made disaster (i.e. human-made disasters without large scale devastations), compared to non-affected residents which were matched on pre-exposure characteristics. The aim of the present study is to fill this gap of scientific knowledge. Because of the absence of (series of) earlier longitudinal studies on the consequences of human-made or technological disasters on wages and employment security in the short (about 1–2 years post-event), medium (3–8 years post-event) and long term (9 years or more post-event), we could not derive empirically based hypotheses. However, given the proven relationship between disasters and mental health on the one side [1–4], and mental health and work or employment and thus wages on the other [9–17], we might expect differences in gross annual wages and employment between affected and non-affected residents. We expected stronger differences in wages and employment security in the short and medium term than in the longer term because in the first time-frame (short-medium) the prevalence of mental health problems and disorders generally reaches its highest levels [1–4, 25, 26]. However, it is also possible that the negative short- and medium-term effects on wages and employment security sustain in the longer term because of scarring effects which prevents people to recover from the adversity (one of the reasons for these long-term scarring effects might be the signaling or screening of people with physical or mental impairments by the employer). In this study, employment security is defined as the number of weeks people are employed during a year (cf. methods section). It is possible that the disaster especially affected vulnerable people such as those with low incomes: we therefore assessed possible differences between subgroups of affected and matched non-affected who worked at the time of the disaster with low annual wage (wages in the lowest two deciles).

For this purpose, we assessed the wage and employment security following the Enschede fireworks disaster in the Netherlands (2000), from 1999 to 2016. This disaster, caused by a massive devastating explosion in a fireworks company (estimated 4.5–5.0 tons of TNT), took place in a residential area in the city of Enschede on May 13, 2000, in the afternoon. Besides the severely damaged and destroyed 500 houses and buildings, 23 persons were killed and about 1,000 residents sustained injuries (mostly because of glass injuries). After this disaster, a large longitudinal health study among the affected residents was conducted up to 10 years post-event. The study was part of the mental health policy of the Dutch government, aimed at offering social, psychological and practical support and advice to the affected residents. In short, results of these earlier studies showed that mental health problems and posttraumatic stress symptoms did follow a relatively “normal” pattern compared to other disasters [27]. For instance, after 2–3 weeks, 1.5 years, 4 years and 10 years post-event 70.0% (95% CI 67.2–72.7), 39.1% (95% CI 36.2–42.1), 26.4% (95% CI 23.6–29.2) and 16.7% (95% CI 14.2–19.1) respectively reported high levels of PTSD symptomatology among affected Dutch native residents [28].

The present study focused on the following research questions. After the Enschede fireworks disaster:

1. To what extent did the gross annual wages of the affected residents differ from the gross annual wages of matched non-affected residents in the period 2000–2017?
2. To what extent did gross annual wages of the affected residents with low annual wages before the disaster differ from gross annual wages of matched non-affected residents in this period?
3. To what extent did the employment security of the affected residents differ from the employment security of matched non-affected residents in this 17-year period?
4. To what extent did the employment security of the affected residents with low annual wages in 1999 differ from the employment security of matched non-affected residents with low annual wages in this period?

## Material and methods

### Data statistics Netherlands

To answer the research questions of the present study, a quasi-experimental design has been chosen in which we compare the development of wage and employment security of affected residents with those of matched non-affected control groups over a period of 17 years. For this purpose, we retrieved the information on wages and employment from Statistics Netherlands microdata services (in Dutch: Centraal Bureau voor de Statistiek (CBS), see www.cbs.nl/microdata). Statistics Netherlands records all individual demographic, income, labor market and household data, including transitions, of the entire Dutch population digitally since 1999 (15.8 million residents in 1999). Access to the microdata of the CBS is granted to scientific researchers under specific and strict privacy securing conditions. According to CBS the following organizations may be granted access to CBS microdata: Dutch universities, institutes for scientific research, organizations for policy advice or policy analysis, statistical authorities in other EU countries, other research institutions authorized to work with the microdata. The third party (CBS) data can be obtained by sending a request to CBS for using the data for academic research by submitting the required application form that can be retrieved from the website: https://www.cbs.nl/en-gb/our-services/customised-services-microdata/microdata-conducting-your-own-research/applying-for-access-to-microdata.

After receiving approval, we analyzed the data through a remote access facility that connected our computers to the protected ICT environment of Statistics Netherlands. Data on income, employment and demographics are linked by using the fiscal number of Dutch inhabitants. After completing the analyses, Statistics Netherlands performed a check on the results to secure anonymity and privacy, i.e. that individual persons cannot be identified (not on the content of the findings). We combined and analyzed various administrative datasets of Statistic Netherlands (variables between brackets): i.e. SECMBUS (main source of income), GBAHUISHOUDENSBUS (household position and children), HOOGSTEOPLTAB (education attainment), BAANSOMMENTAB (annual wage), GBAADRESOBJECTBUS (residential area), GBAPERSOONSTAB (age, gender, and ethnicity).

### Affected and non-affected residents

Based on a list of all postal codes of the affected residential area (see S1 Appendix Postal codes), we were able to identify all individual residents who lived in this area on May 13, 2000 according to the official municipal registers. This means that all individual residents who left the affected area or moved to another place in the Netherlands could be followed.

The massive fireworks explosion took place in a residential area and about 500 houses were destructed or very severely damaged (this area is called “Inner ring”). We subsequently drew a randomly pairwise matched control group from individual residents out of the entire population of Dutch residents and a separate random pairwise matched control group from individual residents from the city of Tilburg in the Netherlands. The city of Tilburg was chosen for several reasons, besides the fact that Tilburg was not affected by the disaster because it is located in another part of the Netherlands. Importantly, Enschede and Tilburg share a similar social-economic history of the rise and fall of textile and metal industries throughout the 1960s up to the early 1980s that was succeeded in both cities by the rise of public and commercial services. In contrast to for example Eindhoven that also had a textile industry, both cities did not harbor a multinational like Philips. Furthermore, in both cities a university is located (of the 13 cities with universities in the Netherlands) and both cities are situated near the border, i.e. the border with Germany and Belgium respectively (relevant for cross-border labor commuting and availability of employment). Of the residents of the city of Enschede (about 150,000 residents in 2000) and Tilburg (about 194,000 residents in 2000) a considerable minority had a migration background (24.7% and 19.7% respectively in 2000). The unemployment rates in the years before the disaster (1996–1998) were equal and high in both cities, i.e. 8.4%. At the time of the disaster a military airbase was situated near both cities. Both cities are part of the Dutch G40-network of (medium-large) cities that share mutual urban affairs with respect to for instance housing, well-being, urban development, employment, and security. Finally, in previous research following this disaster, residents from Tilburg were also chosen as control group, i.e. residents from a residential area in Tilburg with similar characteristics as the affected area in Enschede [27, 28].

### Matching procedures

For the pairwise matching we used a set of seven indicators namely gender, age category, (non-) native, skill/education level (if known), household position, main source of income (socio-economic status), and the position in the decile distribution of the gross annual wage at the day of the disaster. The wage indicator refers to the year 1999 since the gross annual wage of 2000 could already be influenced by the disaster. For information about the categories of each matching variable, we refer to Table 1. The matching was conducted with the k2k option of Coarsened Exact Matching (CEM) [29], allowing us to exactly match individuals on relevant (categorical) characteristics. The CEM procedure creates one stratum for each unique observation of the vector *X* that consisted of the aforementioned set of indicators. For the matching procedure, we have created a preliminary dataset that holds all residents’ statuses at the day of the disaster.

**Table 1:** Demographics of the total group of affected residents (N = 4,216) and affected low-wage group (N = 552) on May 13, 2000.

	Total group		Low-wage group	
	N	(%)	N	(%)
**Sex**				
Male	2,172	(51.52)	272	(49.28)
Female	2,044	(48.48)	280	(50.72)
**Ethnicity**				
Native	3,068	(72.77)	420	(76.09)
Non-native	1,148	(27.23)	132	(23.91)
**Education level**				
Low	780	(18.50)	66	(11.96)
Medium	486	(11.53)	204	(36.96)
High	211	(5.00)	55	(9.96)
Unknown	2,739	(64.97)	227	(41.12)
**Age category (in years)**				
0–16	708	(16.79)	16	(2.90)
17–22	469	(11.12)	282	(51.09)
23–34	1,030	(24.43)	168	(30.43)
35–49	831	(19.71)	56	(10.14)
50–64	654	(15.51)	27	(4.89)
65+	524	(12.43)	3	(0.54)
**Socio-economic status (SES)**				
Working	1,601	(37.97)	114	(20.65)
Unemployment benefits	21	(0.50)	1	(0.18)
Welfare benefits	214	(5.08)	24	(4.35)
Disability benefits	276	(6.55)	24	(4.35)
Retirement benefits	559	(13.26)	7	(1.27)
Full-time student	1,167	(27.68)	338	(61.23)
Not working or entitled to benefits	378	(8.97)	44	(7.97)
**Household position**				
Single	995	(23.60)	229	(41.49)
Partner without children	1,070	(25.38)	94	(17.03)
Partner with children (<5 yrs.)	314	(7.45)	20	(3.62)
Partner with children (≥5 yrs.)	568	(13.47)	36	(6.52)
Lone parent (<5 yrs.)	16	(0.38)	1	(0.18)
Lone parent (≥5 yrs.)	78	(1.85)	8	(1.45)
Child living at parents’ home	979	(23.22)	140	(25.36)
Other	196	(4.65)	24	(4.35)
**Gross annual wage decile [wage in euros x1000] in 1999**				
No wage	2,194	(52.04)	n.a	n.a
>0–1.629	283	(6.71)	284	(51.45)
1.630–4.138	225	(5.34)	268	(48.6)
4.139–8.230	178	(4.22)	n.a	n.a
8.231–12.769	184	(4.36)	n.a	n.a
12.770–17.037	188	(4.46)	n.a	n.a
17.038–20.874	220	(5.22)	n.a	n.a
20.875–24.625	203	(4.81)	n.a	n.a
24.626–28.887	214	(5.08)	n.a	n.a
28.888–36.629	181	(4.29)	n.a	n.a
36.630 and higher	146	(3.46)	n.a	n.a

Note: Skill level is based on the International Standard Classification of Education (1-digit ISCED 2011). Skill level is divided into [0] ‘Low skilled’, and includes the ISCED levels 0 to 2; [1] ‘Medium skilled’ includes the ISCED levels 3 to 4; and [2] ‘High skilled’ for ISCED levels 5 to 8. n.a. = not applicable because of the selection.

Unlike the 100 percent matching of the affected residents with the control group drawn from the general Dutch population, due to smaller sample sizes, not all affected residents could be matched with residents in the chosen areas of the city of Tilburg. In the next step we therefore tried to match them with residents of other residential areas of the city of Tilburg. After this selection process in total 97.1% of all affected residents was matched.

The total sample size in the present study (N = 12,648) includes 4,216 affected residents and 2x 4,216 pairwise matched residents from Tilburg and the Netherlands in the year 2000 (see Table 1).

### Measures

The dependent variables in our study are annual wages and employment security as measured over the period 1999 to 2016. Employment security is measured by the number of weeks individual residents were employment secure during a particular year, regardless of their working hours during the week. The wage is the gross annual wage before taxes. Since we analyze annual wages and employment security of people for over 17 years, we actually study wage and employment security.

The variable socio-economic status (SES) of the neighborhood individual residents were living in during (most of) the year was based on the percentage of residents with: a low education, a migration background, a low gross annual wage (a wage in the two lowest deciles), and receiving benefits (unemployment, illness, working disabilities). To arrive at a score on neighborhood SES, the percentages were averaged and indexed between 0 and 100. The identification of the neighborhood itself is derived from Statistics Netherlands, which has created specific neighborhood codes (‘Buurtcode’ in Dutch) for each Dutch city.

The low-wage group was defined as the affected residents (and their matched counterparts) that belong to the lowest two deciles of the wage distribution (lowest 20%) using the gross annual wage of 1999 (individuals without a wage are not included). We decided not to observe the post-disaster effect of those without earnings from paid work in 1999. It can be argued that the low-wage group is more prone to job loss given the aforesaid reasons since the employment rate in this neighborhood was already low by default.

#### Statistical analyses

Fixed-effects linear panel regression models on gross annual wages and employment security were estimated to assess both the development in the affected group of residents compared to those of the two control groups in the period 1999–2016. The same analyses were conducted among the low-wage group since this group may be more vulnerable. Fixed-effects models are appropriate for studying the causal effects while they allow to correct for the heterogeneity associated with time invariant unobserved individual characteristics not available in the administrative data (for instance intelligence, DNA, ethnicity) and which might confound the career outcomes. Because we focus on wages and employment, respondents who were younger than 16 years or were 65 years or older in a particular year, were excluded from the analyses. However, the youngest group becomes included in the analyses as soon as they turn 16 years of age. In the pairwise matching procedure, we already controlled for education level, main source of income, and position in the household represented by the vector *X*.

In this way, we are able to assess the effects of the disaster on the victims’ wage and employment Eq 1, employment security, E_it_, is defined in this study as the number of weeks people are employed during a year, varying between 0 and 52 [30]. Alternately, gross annual wage, W_it_, is the gross annual income in euros earned through paid labor. Since we analyze annual wages and the employment security of people for over a period of 17 years, we actually study the short, medium as well as the long-term effects of the disaster.

The impact of the disaster on the development of annual wages and employment security is indicated by a full set of interaction dummies of year, YEAR_t_, which is interacted with each corresponding control group (i.e., Tilburg and the Netherlands) represented by respectively DTil_i_ and DNL_i;_ affected residents are the reference category in each analysis. The estimates on the interaction terms on both employment security and gross annual wages indicate to what extent the control groups performed better or worse in relation to affected residents of the disaster area.

Furthermore, we added some additional controls indicated by C_it_ where we controlled for the SES of the neighborhood each respondent was living in during a particular year (or the largest part of the year; SES_it_) since it may impact gross annual wages and employment [6, 30]. Neighborhood’s SES is a time-variant variable because of changes in its socio-economic composition, e.g. caused by people moving to another dwelling in another area (the same or another city). Neighborhood’s SES serves as an indicator of people’s social capital measured in this paper by the socio-economic conditions of the people living in the neighborhood. It is well-known from the sociological literature that the higher the level of neighborhood social capital, the more likely it is that people get appropriate job offers [31–35]. In addition, C_it_ also controls for changes in the household position interacting with gender (HH_it_*GDNDR_i_), and for changes in the educational level (EDU_it_) in the post-disaster years. In the case of significant differences between the affected and non-affected citizens each year, we computed the Cohen’s d measure to calculate the effect size (Eq 2), that is, the strength of the difference in the predicted values in that year. The statistical analyses were conducted with STATA version 14.

$$E_{\mathrm{it}}{/W}_{\mathrm{it}}{=\beta }_{0}+\overset{T}{\underset{t=1999}{\sum }}\beta_{t}{Year}_{t}{+\delta }_{1}{\mathrm{DTil}}_{i}\overset{T}{\underset{t=1999}{\sum }}{Y\mathrm{EAR}}_{t}{+\delta }_{2}{\mathrm{DNL}}_{i}\overset{T}{\underset{t=1999}{\sum }}{Y\mathrm{EAR}}_{t}{+\gamma }_{1}C_{\mathrm{it}}{+\mu }_{i}{+\varepsilon }_{\mathrm{it}}$$ (1)

$$\mathrm{where}\;C_{\mathrm{it}}=\chi_{1}{\mathrm{SES}}_{\mathrm{it}}+\chi_{2}{E\mathrm{DU}}_{\mathrm{it}}+\chi_{3}{\mathrm{HH}}_{\mathrm{it}}\cdot {G\mathrm{NDR}}_{i}$$

$$d=\frac{M_{\mathrm{treat}}{‑M}_{\mathrm{control}}}{\sqrt{[}{(\mathrm{sd}}_{{\mathrm{treat}}^{2}}+{\mathrm{sd}}_{{\mathrm{control}}^{2}})/2]}$$ (2)

We did not use Tobit because this approach cannot be used in combination with fixed effects due to the large number of included residents. If we want to correct for fixed effects, we must include personal identifiers as dummy variables (n-1) creating about 12,000 dummy variables (cf. www.princeton.edu/~otorres/Panel101.pdf). Importantly, the error terms of predicted values appeared to be normally distributed.

#### Ethics

For the present study, we used anonymized data from Statistics Netherlands that records on a monthly basis all individual demographic, gross wages, employment and household data of the entire Dutch population since 1999. Because we used administrative data, no medical ethical committee or IRB approval is needed in the Netherlands according to Dutch law. However, as said, Statistics Netherlands performed a check on our results to secure anonymity and privacy, i.e. that individual persons could not be identified.

## Results

### Characteristics affected and non-affected residents

Table 1 shows the characteristics of the total group of affected residents and the low-wage group on May 13, 2000. Because of the pairwise matching procedures, the characteristics of the non-affected residents from the city of Tilburg and from the entire Dutch population are similar and thus not presented in Table 1. Due to retirement or death of residents, and residents reaching 16 years of age in a particular year, the number of respondents each year changes and differs to some extent between study groups and years (see S2 to S5 Appendix).

Table 1 further shows that more than half of the affected residents with low wages, as well as their matched counterparts, consisted of full-time students (61.23%) having perhaps a marginal income. Only 20.65% was registered as a ‘worker’ receiving earnings from paid work. To be clear, all others receive either unemployment benefits, social welfare or disability benefits which are not included in the wage data.

### Differences in gross annual wages between total group of affected residents and comparison groups

**Fig 1:**
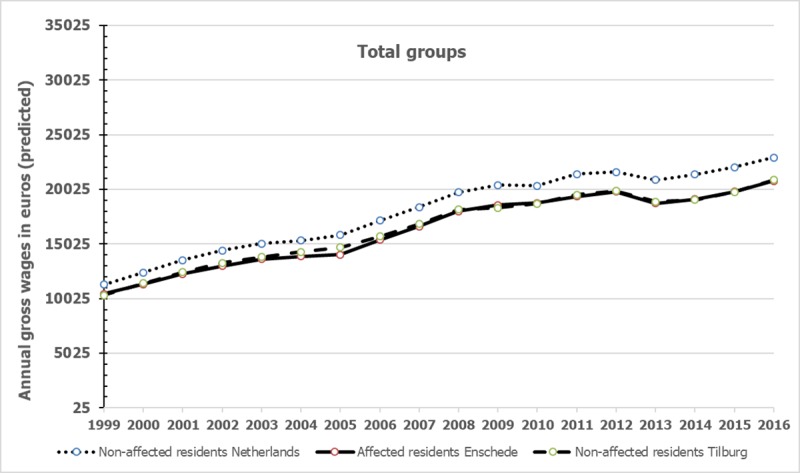
Gross annual mean wages of the total group of affected residents and the non-affected control groups.

The predicted mean gross annual wages of the total groups of affected residents and comparison groups from 1999 to 2016, i.e. wages after taking account of the effects of the control variables, are graphically presented in Fig 1. For the exact observed and predicted means, standard deviations and sample sizes we refer to S2 Appendix. Fig 1 shows that over the years the predicted mean wages of the distinguished groups approximately doubled.

The results of the fixed-effects linear panel regression analyses on annual wages of the total group of affected residents and comparison groups are shown in Table 2. The final statistics are presented at the end of Table 2. The effects of year, control variables and intercept are not shown to prevent lengthy tables. In the case that the predicted annual wages of the groups differed significantly (p < 0.05), the Cohen’s d is presented in the same row.

**Table 2:** Results of fixed-effects linear panel regression models for total groups with gross annual wages as dependent variable (1999–2016).

Affected versus non-affected residents the Netherlands (total groups)						Affected versus non-affected residents Tilburg (total groups)				
Year	b	SE	t	p <	Cohen’s d	b	SE	t	p <	Cohen’s d
1999	(ref.)					(ref.)				
2000	488.14	410.92	1.19	0.24	-	642.13	411.01	1.56	0.12	-
2001	781.23	414.33	1.89	0.06	-	833.21	412.38	2.02	0.04	0.02
2002	933.94	415.67	2.25	0.03	0.22	800.44	413.19	1.94	0.05	-
2003	658.50	414.61	1.59	0.11	-	554.91	415.71	1.33	0.18	-
2004	867.35	416.53	2.08	0.04	0.24	903.83	415.97	2.17	0.03	0.03
2005	1246.35	418.06	2.98	0.00	0.24	1343.20	418.34	3.21	0.00	0.06
2006	1082.04	418.60	2.58	0.01	0.28	985.60	418.95	2.35	0.02	0.10
2007	1103.49	419.17	2.63	0.01	0.25	905.47	419.61	2.16	0.03	0.04
2008	1079.45	419.76	2.57	0.01	0.24	847.25	420.92	2.01	0.04	0.03
2009	1072.32	421.54	2.54	0.01	0.24	368.32	421.34	0.87	0.38	-
2010	914.64	422.49	2.16	0.03	0.24	544.34	422.49	1.29	0.20	-
2011	1289.80	423.19	3.05	0.00	0.20	725.86	423.67	1.71	0.09	-
2012	1017.22	424.09	2.40	0.02	0.26	582.72	424.80	1.37	0.17	-
2013	1385.04	425.83	3.25	0.00	0.22	739.43	426.27	1.73	0.08	-
2014	1595.82	426.92	3.74	0.00	0.26	308.28	427.74	0.72	0.47	-
2015	1433.26	429.64	3.34	0.00	0.28	444.45	429.80	1.03	0.30	-
2016	1327.74	432.46	3.07	0.00	0.26	574.79	432.71	1.33	0.18	-
σ_u_ = 14065.32, p < 0.001										
σ_e_ = 11300.84, p < 0.001										
ρ = 0.61										
F (72, 147012) = 468.69, p <0.001										
R^2^ ^within^ = 0.19										
R^2^ ^between^ = 0.30										
R^2^ ^overall^ = 0.23										
N (Obs) = 158,285										
N (Ind) = 11,201										

Note: Differences between groups were assessed within one analysis. The year 1999 is the reference year and the affected residents of Enschede the reference group. Fixed-effects linear panel regression models with education level, employment security, partner wages, household position, neighborhood’s SES as control variables (data not shown in table). Sigma_u (σ_u)_ = sd of residuals within individuals (entities) u_i_. Sigma_e (σ_e)_ = sd of residuals (overall error term) e_i_. Rho (ρ) = % of the variance is due to differences across panels. ‘rho’ is known as the intraclass correlation. R^2^ within = explained variance within persons over time (entities). R^2^ between = explained variance between persons (entities). R^2^ overall = averaged explained variance. N (Obs) = number of observations (time x ind.). N (Ind) = number of individuals (entities).

Table 2 shows that the matched group of residents from the Netherlands had significant higher (predicted) wages than the affected group of residents in the year 2002 and from the year 2004 on, up to the end of the observation period in 2016. However, the computed Cohen’s d revealed that the differences are all below .30 and therefore rather small. Affected residents also had significant lower wages than the non-affected residents from Tilburg in 2001 and from 2004 up to 2008, but effects sizes were all below 0.10 indicating trivial differences in these years.

### Differences in gross annual wages between affected residents with low wages and comparison groups

**Fig 2:**
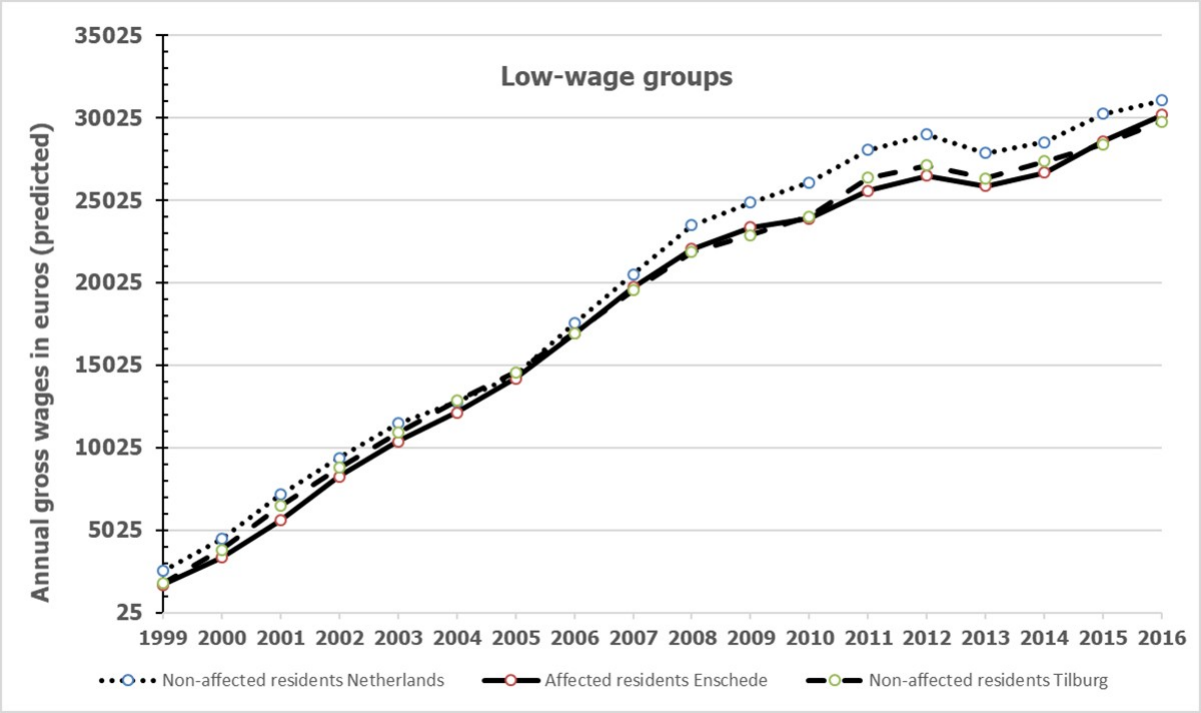
Gross annual mean wage of the low-wage group of affected residents and the non-affected control groups.

The predicted mean gross annual wages among the low-wages groups from 1999 to 2016 are graphically presented in Fig 2, similar to Fig 1. Fig 2 suggests that among the low-wage groups (wages in lower 20% of all wages) in 1999, the mean wages increased at a much higher rate than among the total group of affected residents and comparison groups. However, it is difficult to compare Figs 1 and 2 because Fig 1 contained a large group of unemployed affected and non-affected residents (see Table 1), while Fig 2 shows the predicted mean wages of residents who did work or already worked in 2000.

The results of the fixed-effects linear panel regression analyses on annual wages among the low-wages groups are presented in Table 3 in a similar way as Table 2. In the case that the predicted annual wages of the groups differed significantly (p < 0.05), the Cohen’s d is presented in the same row. Results revealed that among the groups with low annual wages (<20%) in 1999, non-affected residents from the Netherlands had a significant higher income in only 2012 but again the difference was small (Cohen’s d = 0.24). No significant differences between affected residents and the matched low-wage group of residents from Tilburg were found. Notice the increase in the explained variance compared to the model that included the total group, implying that there is some endogeneity in the model when observing the wage career of the low-wage group.

**Table 3:** Results of fixed-effects linear panel regression models for low-wage groups, with gross annual wages as dependent variable (1999–2016).

Affected versus non-affected residents the Netherlands (low-wage groups)						Affected versus non-affected residents Tilburg (low-wage groups)				
Year	b	SE	t	p <	Cohen’s d	b	SE	t	p <	Cohen’s d
1999	(ref.)					(ref.)				
2000	909.16	1060.83	0.86	0.39	-	1528.89	1063.36	1.44	0.15	-
2001	1393.77	1064.38	1.31	0.19	-	2080.59	1063.52	1.96	0.05	-
2002	814.96	1065.33	0.76	0.44	-	1377.43	1064.06	1.29	0.20	-
2003	426.43	1063.63	0.40	0.69	-	1077.55	1067.76	1.01	0.31	-
2004	153.49	1067.32	0.14	0.89	-	1114.90	1068.66	1.04	0.30	-
2005	-271.12	1070.82	-0.25	0.80	-	780.21	1073.75	0.73	0.47	-
2006	-53.93	1072.60	-0.05	0.96	-	116.03	1073.66	0.11	0.91	-
2007	43.35	1074.12	0.04	0.97	-	487.18	1075.94	0.45	0.65	-
2008	735.85	1076.10	0.68	0.49	-	732.39	1079.41	0.68	0.50	-
2009	299.67	1080.63	0.28	0.78	-	269.22	1081.64	0.25	0.80	-
2010	1375.61	1083.05	1.27	0.20	-	680.44	1084.70	0.63	0.53	-
2011	1881.37	1085.18	1.73	0.08	-	974.77	1087.62	0.90	0.37	-
2012	2203.09	1088.91	2.02	0.04	0.24	985.03	1091.20	0.90	0.37	-
2013	2038.75	1091.89	1.87	0.06	-	986.44	1095.57	0.90	0.37	-
2014	1663.30	1097.64	1.52	0.13	-	746.48	1100.60	0.68	0.50	-
2015	1242.20	1103.70	1.13	0.26	-	-272.58	1105.62	-0.25	0.81	-
2016	477.59	1108.78	0.43	0.67	-	-348.38	1109.26	-0.31	0.75	-
σ_u_ = 12297.88, p < 0.001										
σ_e_ = 12297.88, p < 0.001										
ρ = 0.46										
F (72, 26034) = 277.61, p < 0.001										
R^2 within^ = 0.43										
R^2 between^ = 0.39										
R^2 overall^ = 0.40										
N (Obs) = 27,810										
N (Ind) = 1,649										

Note: Differences between groups were assessed within one analysis. The year 1999 is the reference year and the affected residents of Enschede the reference group. Fixed-effects linear panel regression models with education level, employment security, partner wages, household position, neighborhood’s SES as control variables (data not shown in table). Sigma_u (σ_u)_ = sd of residuals within individuals (entities) u_i_. Sigma_e (σ_e)_ = sd of residuals (overall error term) e_i_. Rho (ρ) = % of the variance is due to differences across panels. ‘rho’ is known as the intraclass correlation. R^2^ within = explained variance within persons over time (entities). R^2^ between = explained variance between persons (entities). R^2^ overall = averaged explained variance. N (Obs) = number of observations (time x ind.). N (Ind) = number of individuals (entities).

Furthermore, the increases in the standard deviations of the predicted wage level compared to those of the actual wage in 1999 (see S3 Appendix) are due to the unobserved heterogeneity part of the fixed-effect model. This has been investigated through dropping each of the observed control variables which however did not change the explained variance nor the standard deviations much. Hence, the unobserved heterogeneity part of the model must have caused it. This can be shown by predicting the unobserved variance (the within-subject residuals) showing that for the low-wage group there are significant differences in the means and standard deviations of wages across the various treatment groups (*F* (2,158282) = 68.92, p<0.000).

### Differences in employment security between total group of affected residents and comparison groups

**Fig 3:**
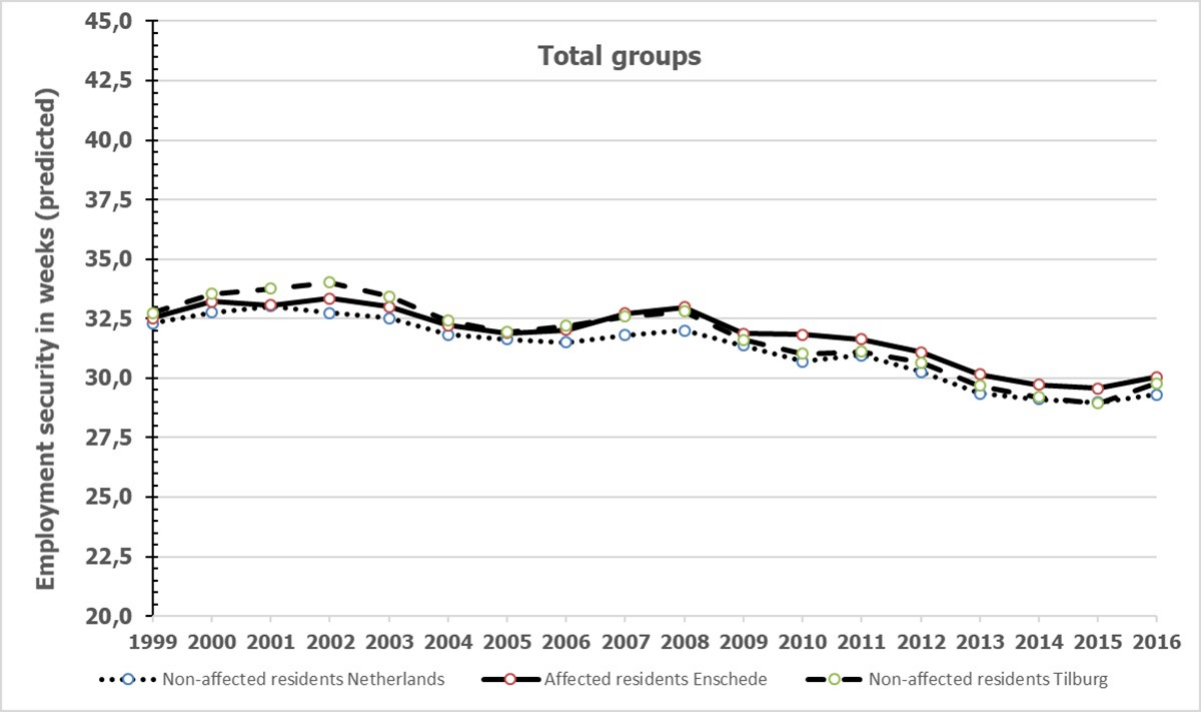
Annual employment security of the total group of affected residents and non-affected control groups.

Employment security among the total group of affected residents and comparison groups in the period 1999–2016 are represented in Fig 3, similar to Fig 1. For information on the observed and predicted means, standard deviations and numbers we refer to S4 Appendix. Fig 3 shows that the employment security follows a similar pattern across the total group of affected residents, the comparison group from the Netherlands and the comparison group from Tilburg with low wages.

The results of the regression analyses with employment security as dependent variable are presented in Table 4. Analyses among the total group did not show that affected residents were less employed over the years than non-affected residents from the Netherlands and Tilburg, besides some significant differences between affected and matched control from the Netherlands that were trivial given the low Cohen’s d of ≤ 0.10 (2007 and 2010).

**Table 4:** Results of fixed-effects linear panel regression models for total groups with employment security as dependent variable (1999–2016).

Affected versus non-affected Residents the Netherlands (total groups)						Affected versus non-affected residents Tilburg (total groups)				
Year	b	SE	t	p <	Cohen’s d	b	SE	t	p <	Cohen’s d
1999	(ref.)					(ref.)				
2000	0.37	0.52	-0.72	0.47	-	0.09	0.52	0.18	0.86	-
2001	-0.24	0.52	-0.46	0.65	-	0.47	0.52	0.90	0.37	-
2002	-0.82	0.52	-1.58	0.12	-	0.61	0.52	1.18	0.24	-
2003	-0.30	0.52	-0.58	0.56	-	0.65	0.52	1.24	0.21	-
2004	-0.49	0.52	-0.94	0.35	-	0.20	0.52	0.39	0.70	-
2005	-0.35	0.53	-0.67	0.50	-	-0.26	0.53	-0.49	0.62	-
2006	-0.70	0.53	-1.32	0.19	-	-0.03	0.53	-0.06	0.96	-
2007	-1.06	0.53	-2.01	0.04	-0.10	-0.21	0.53	-0.39	0.70	-
2008	-1.01	0.53	-1.90	0.06	-	-0.10	0.53	-0.18	0.86	-
2009	0.78	0.53	-1.47	0.14	-	-0.29	0.53	-0.55	0.58	-
2010	-1.30	0.53	-2.45	0.01	-0.09	-0.80	0.53	-1.50	0.13	-
2011	-0.86	0.53	-1.62	0.11	-	-0.58	0.53	-1.09	0.28	-
2012	-0.95	0.53	-1.78	0.08	-	-0.52	0.53	-0.97	0.33	-
2013	-1.04	0.54	-1.93	0.05	-		-0.70	0.54	-1.30	0.20	-
2014	-0.70	0.54	-1.31	0.19	-	-0.59	0.54	-1.09	0.28	-
2015	0.91	0.54	-1.68	0.09	-	-0.76	0.54	-1.40	0.16	-
2016	-1.12	0.54	-2.06	0.04	-	-0.67	0.54	-1.23	0.22	-
σ_u_ = 19.84, p < 0.001										
σ_e_ = 14.23, p < 0.001										
ρ = 0.66										
F(72, 147012) = 78.31, p < 0.001										
R^2^ ^within^ = 0.04										
R^2^ ^between^ = 0.03										
R^2 overall^ = 0.05										
N (Obs) = 158,285										
N (Ind) = 11,201										

Note: Differences between groups were assessed within one analysis. The year 1999 is the reference year and the affected residents of Enschede the reference group. Fixed-effects linear panel regression models with education level, employment security, partner wages, household position, neighborhood’s SES as control variables (data not shown in table). Sigma_u (σ_u)_ = sd of residuals within individuals (entities) u_i_. Sigma_e (σ_e)_ = sd of residuals (overall error term) e_i_. Rho (ρ) = % of the variance is due to differences across panels. ‘rho’ is known as the intraclass correlation. R^2^ within = explained variance within persons over time (entities). R^2^ between = explained variance between persons (entities). R^2^ overall = averaged explained variance. N (Obs) = number of observations (time x ind.). N (Ind) = number of persons (entities).

### Differences in employment security between affected residents with low wages and comparison groups

**Fig 4:**
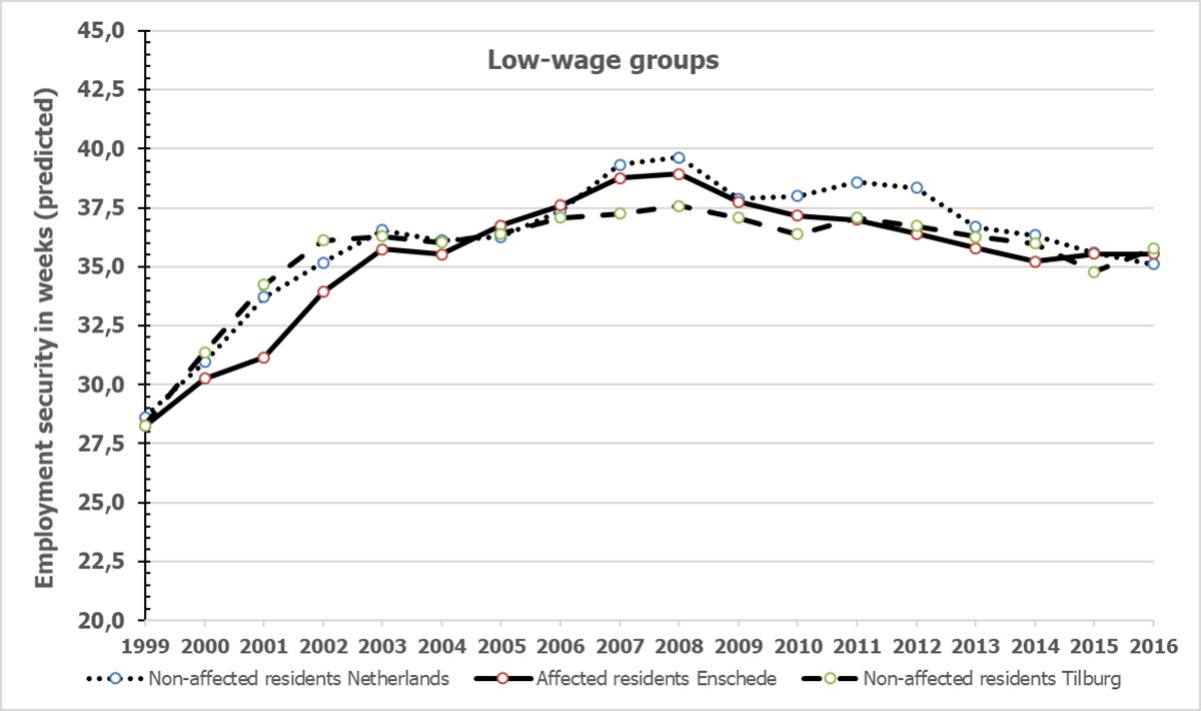
Annual employment security of affected residents with low wages and non-affected control groups.

The employment security on the affected residents with low wages and comparison groups in the period 1999–2016 are represented in Fig 4, similar to Fig 1. For information on the observed and predicted means, standard deviations and numbers we refer to S5 Appendix. Fig 3 shows that the employment security among the affected residents with low wages does not follow the pattern of the comparison groups from the Netherlands and from Tilburg with low wages.

Table 5 shows that according to the fixed-effects linear panel regression models the employment security of affected residents with low wages did not differ from the comparison groups from the Netherlands with low wages, besides a trivial difference in 2001 (see Table 5). However, for the years 2001 and 2002, affected residents appeared to be significantly fewer weeks employed than the matched non-affected residents from Tilburg. Only for 2002 the difference between these two groups appeared to be above moderate (Cohen’s d = 0.68) indicating that in 2002 affected residents were fewer weeks employed than non-affected from Tilburg.

**Table 5:** Results of fixed-effects linear panel regression models for low-wages groups with employment security as dependent variable (1999–2016).

Affected versus non-affected residents the Netherlands (low-wage groups)						Affected versus non-affected residents Tilburg (low-wage groups)				
Year	b	SE	t	p <	Cohen’s d	b	SE	t	p <	Cohen’s d
1999	(ref.)					(ref.)				
2000	0.75	1.29	0.58	0.56	-	1.78	1.30	1.37	0.17	-
2001	2.63	1.30	2.02	0.04	0.18	3.81	1.30	2.93	0.00	0.30
2002	1.22	1.30	0.94	0.35	-	2.84	1.30	2.19	0.03	0.68
2003	0.56	1.30	0.43	0.67	-	0.97	1.30	0.74	0.46	-
2004	0.26	1.30	0.20	0.84	-	0.87	1.30	0.67	0.50	-
2005	-0.77	1.31	-0.59	0.56	-	-0.02	1.31	-0.01	0.99	-
2006	-0.59	1.31	-0.45	0.65	-	-0.44	1.31	-0.34	0.74	-
2007	0.23	1.31	0.17	0.86	-	-1.05	1.31	-0.80	0.42	-
2008	0.36	1.31	0.28	0.78	-	-0.84	1.32	-0.64	0.52	-
2009	-0.42	1.32	-0.32	0.75	-	-0.42	1.32	-0.32	0.75	-
2010	0.44	1.32	0.33	0.74	-	-0.55	1.32	-0.41	0.68	-
2011	1.33	1.32	1.00	0.32	-	0.09	1.33	0.07	0.95	-
2012	1.69	1.33	1.27	0.20	-	0.44	1.33	0.33	0.74	-
2013	0.78	1.33	0.59	0.56	-		0.65	1.34	0.49	0.63	-
2014	0.89	1.34	0.66	0.51	-	0.73	1.34	0.54	0.59	-
2015	-0.15	1.35	-0.11	0.91	-	-0.75	1.35	-0.56	0.58	-
2016	-0.56	1.35	-0.41	0.68	-	0.41	1.35	0.30	0.76	-
σ_u_ = 15.02, p < 0.001										
σe = 15.01, p < 0.001										
ρ = 0.50										
F (72, 26034) = 27.59, p < 0.001										
R^2 within^ = 0.07										
R^2 between^ = 0.11										
R^2 overall^ = 0.09										
N (Obs) = 27,755										
N (Ind) = 1,649										

Note: Differences between groups were assessed within one analysis. The year 1999 is the reference year and the affected residents of Enschede the reference group. Fixed-effects linear panel regression models with education level, employment security, partner wages, household position, neighborhood’s SES as control variables (data not shown in table). Sigma_u (σ_u)_ = sd of residuals within individuals (entities) u_i_. Sigma_e (σ_e)_ = sd of residuals (overall error term) e_i_. Rho (ρ) = % of the variance is due to differences across panels. ‘rho’ is known as the intraclass correlation. R^2^ within = explained variance within persons over time (entities). R^2^ between = explained variance between persons (entities). R^2^ overall = averaged explained variance. N (Obs) = number of observations (time x ind.). N (Ind) = number of persons (entities).

## Discussion

To the best of our knowledge, this is the first longitudinal study examining the wage and employment security of residents affected by a major human-made technological disaster using a quasi-experimental design with matched comparison groups of non-affected residents over a period of 17 years.

We found that the total group of affected residents compared to matched non-affected residents from the Netherlands had significant lower annual wages over a period of about 14 years starting two years post-event, but the differences were small according to Cohen’s d. We found no indications that the differences became larger over the years. The differences in gross annual wages between affected residents and matched non-affected residents from Tilburg, a city with a comparable historical background, were trivial. In addition, we found no indications that among the low-wage group, affected residents had lower wages up to 16 years post-event than the non-affected comparison low-wage groups in 1999.

With respect to employment security among the total group, no differences were found between the affected residents and non-affected residents of Tilburg. Only in 2007 and 2010 affected residents differed significantly from the non-affected residents from the Netherlands. The last group was fewer weeks employed than the affected group in these years, but the differences were trivial again. A comparison with the non-affected residents from Tilburg showed no significant differences. Analyses among the residents with low incomes in 1999 showed no relevant differences between affected and non-affected residents from the Netherlands. However, compared to the matched non-affected Tilburg residents, affected residents were in 2002 clearly fewer weeks employed: the differences were above moderate according to Cohen’s d. The employment security of low-wage affected residents did not differ in 2002 from the non-affected group from the Netherlands. Affected low-wage residents had a somewhat lower employment record in 2002 than non-affected low-wage sampled residents from Tilburg and the Netherlands. Remarkably, no differences were found in wages in this year. In sum, we found no evidence that gross annual wages and employment were systematically and substantially lower among the affected residents than among the matched control groups over the years from 2000 up to 2016.

The finding that employment security was not worse among the total group of affected residents than among the control groups seems partly in line with the results of earlier studies. Two examples are the study of Searing and her colleagues [36] among victims of the conflict in Bosnia and Herzegovina (1992–1995) and the study of Vogt and her colleagues [37] among US veterans. Searing and her colleagues (2013) concluded “*This study provides some evidence that not only does time decrease the frequency of mental preoccupation with conflict*, *but that individuals who have endured significant trauma on a PTSD-type scale can succeed in their reintegration with normal economic life* (page 172)”. Vogt and colleagues [37] found that unemployment rates among US male veterans were not higher but lower than among the larger population in the previous two years (2.2% versus 4.8%) and concluded “*These findings speak to the resilience of our service members*, *a topic that has received too little attention in the broader national conversation about veteran readjustment* (p. 349)”. However, in the study of Matthews [12] among road-accident survivors and the study of Savoca and Rosenheck [14] among Vietnam-era veterans, those with PTSD compared to those without PTSD had lower employment and earned less. These different outcomes seem contradictory at first sight, but are less contradictory when differences in study designs are taken into account. The second study compares the outcomes within the veteran’s population (veterans with PTSD versus veterans without PTSD) whereas the first study compares veterans with the rest of the US population. In the general population, which is also one of our control groups, there are always subgroups with mental health problems or subgroups suffering from other problems that may impact their annual wages and employment apart from the effects of potentially traumatic events which need to be sorted out.

In either way, results of Searing [36] and Vogt [37] and colleagues, but also our findings show that resilience should not only be viewed in terms of (the absence of) mental health or mental health problems, as is very often the case. This leaves aside the discussion in the literature on how to define resilience as a process (such as the interaction between stress resistance and stress recovery), outcome (such as the absence of mental health problems) or a personal characteristic (such as personal capacity) [37, 38]. As Britt and colleagues [38] wrote and questioned “*will the same individuals be identified as resilient in the aftermath of adversity when looking at* (for instance*) job performance as the criterion versus mental health*? (p. 396)”. In other words: although victims of human-made disaster are more at risk with respect to mental health problems on especially the short to medium term, they are not more at risk with respect to their annual wages and employment security. This elucidates that resilience must be considered a multilayered phenomenon and should include, besides work outcomes (such as on employment and wages) other domains of life and work [38–43]. Only in 2002, affected low-income residents in 1999 were less employed than non-affected low-income residents from Tilburg in 2002.

Besides the resilience of the affected residents, the help and support offered and provided by others may also help to explain our findings. As mentioned earlier, after the fireworks disaster the Dutch government developed a mental-health policy, including the aforementioned 4-wave study, a municipal Advise and Information Centre (AIC), special Mental Health Services facility (Mediant nazorg) to support and offer treatment to affected residents (and rescue workers) with mental health problems for the first four-five years post-event, and (partial) financial compensations for residents and affected companies. In addition, many other organizations actively supported the affected residents, including the representative organization of affected residents (BSVE), insurance companies, housing cooperations, residents’ employers, and the municipality of Enschede. Without neglecting the prevalence of severe post-event mental health problems during the years afterwards, we assume that all these activities enhanced the resilience and coping-self efficacy of the affected residents [44, 45]. The way health care and social welfare are organized in the Netherlands may have contributed to our results too. In sum, aforementioned activities incorporated Hobfoll’s and colleagues [46] description of important elements of immediate and mid–term mass trauma intervention, i.e. promoting a sense of safety, calming, a sense of self–and community efficacy, connectedness, and hope.

### Contrasting findings

However, while employment security among the total group of affected residents did not differ from employment security among the non-affected residents from the Netherlands, it is unclear why the annual wages were systematically and significantly lower among the affected in the medium and long run. One possible explanation is that this disaster eventually affected residents appreciation of one's life, i.e. leads to different value judgement of specific domains of life (such as “I realize now that my family and children are most important for me” [43], and therefore lower the importance attached to other domains of life such as one’s ambitions at work (i.e. obtain higher wages). In Matthews’ study [12] among road accident victims, respondents with PTSD reported significantly greater extrinsic motivation to work than those without PTSD. In the past years, an increasing number of studies examined so-called posttraumatic growth following potentially traumatic events [47–49], and appreciation of one's life is considered an important aspect of this phenomenon [50, 51]. In their meta-analyses, Shakespeare and Lurie-Beck [52] showed that there is a stronger curvilinear relationship instead of a linear relationship between posttraumatic growth and PTSD symptomatology. Future research should further examine this causality, i.e. to what extent these events affect the work ambitions in the medium and long run among disaster victims. However, we should keep in mind that the differences in annual wages with the non-affected from the Netherlands are shown to be small and hardly differed from the non-affected from Tilburg.

Our findings seem to differ from the Lehman and Wadsworth [24] study following the Chernobyl disaster. However, this major disaster, like major floodings, affected and affected a very large area in contrast to the Enschede fireworks disaster which destructed a residential area in only one residential area of one city which may complicate comparisons.

### Limitations, strength and future directions

In the 4-wave Enschede fireworks disaster study [27], the mental health of the affected residents was assessed. Unfortunately, it was impossible to combine these mental health data with the socio-economic data of Statistics Netherlands that would have enriched the present study. For this reason, we could not assess the wages and employment security among those with severe or persistent mental health problems or PTSD symptomatology compared to affected residents without these problems [12]. We have no data on sickness leave or absenteeism that may differ between affected and non-affected residents, especially during the first day, weeks or months. Furthermore, we could not examine to what extent affected residents with mental health problems differ in annual wages and employment security from non-affected residents with similar problems. Due to the destruction of houses many residents had to be (temporarily) relocated. Hikichi and colleagues [6] showed that group relocation, as compared to individual relocation, appeared to preserve social participation and informal socializing in the community that may affect health and well-being on their turn. Unfortunately, we have no information on group versus individual relocation, and other variables that may affect wages and employment security such as social support for employers and colleagues. In total, 23 persons were killed in the Enschede fireworks disaster. Due to privacy regulations we could not examine the wages and employment security of the bereaved families in the study period. It was outside the aim of the present study to assess possible differences between males and females.

Our longitudinal comparative quasi-experimental study design and collected data of all individual affected and non-affected residents over the years contribute to the methodological strength of the present study. In contrast to the usual post-disaster studies among affected residents who participate in surveys, including studies with clinical interviews, non-response and possible response bias is not present in our study. Furthermore, we were able to match control groups based on the situation just before disaster exposure, which allowed us to assess whether developments after the disaster were different from non-affected individuals with a similar starting position. The inclusion of control groups consisting of pairwise matched individual residents from the city of Tilburg and from the Netherlands as a whole is another important strength, because it enabled us to better understand the differences in gross annual wages. Without the control group of Tilburg, the effects of the disaster on gross annual wages, based on comparisons with the control group of the Netherlands, may be overestimated. In other words, the inclusion of the control group of the Netherlands indirectly but clearly demonstrates that the choice for a particular control group may influence study outcomes. Importantly, in contrast to many other studies we were able to control for the objective social characteristics of the neighborhood. In addition, the disaster took place in a Western country: results may not be applicable to developing or low-income countries or man-made disaster that destruct large areas, complete infrastructures and industries.

### Conclusions

Nevertheless, the results of this novel 17-year longitudinal comparative disaster study can be considered hopeful. Results show that it is not a *conditio sine qua non* that victims of similar human-made disasters have to deal and cope with much lower wages and employment rates in the short, medium and long term although they do suffer from post-event mental health and posttraumatic stress symptoms according to other research [27, 28]. Furthermore, findings demonstrate that the resilience of affected residents should not only be viewed as the relative absence of post-event mental health problems. The question to what extent the provided support from local welfare and health authorities, housing cooperations, insurance companies et cetera exactly contributed to our findings is highly relevant but very difficult to examine. However, findings seem to suggest that when, due to a similar disaster gross annual wages and employment are substantial negatively affected, there is room for improvement for these organizations.

## Supporting information

S1 Appendix Postal codes. (DOCX)

S2 Appendix Gross annual wages affected residents and non-affected controls groups. (DOCX)

S3 Appendix Gross annual wages affected residents and controls groups with low wages in 1999. (DOCX)

S4 Appendix Employment security affected residents and non-affected controls groups. (DOCX)

S5 Appendix Employment security affected residents and non-affected controls groups with low wages in 1999. (DOCX)
