# Supplementary material for: Wages and employment security following a major disaster: A 17-year population-based longitudinal comparative study
Source: PLoS One. 2019 Mar 29;14(3):e0214208. doi: 10.1371/journal.pone.0214208 (PMC6440641; doi:10.1371/journal.pone.0214208)
Supplement: S1 Appendix — (DOCX) [file pone.0214208.s001.docx]

POSTAL CODES

7514AP

7514BM

7514BN

7514BP

7514BR

7514BS

7514BT

7514CJ

7514CR

7514CS

7514CT

7514CV

7522BG

7522BH

7522BL

7522BM

7522BT

7522BV

7522BW

7522BX

7522CA

7522CB

7522CC

7522CD

7522CG

7522CH

7522CJ

7522CK

7522CL

7522CM

7522CN

7522EP

7522ER

7522ES

7522ET

7522EV

7522EW

7522EX

7522EZ

7522GA

7522GB

7522GC

7522GD

7522GE

7522GH

7522GJ

7522RA

7522RB

7523AA

7523AD

7523AP

7523AR

7523AS

7523AT

7523AV

7523AW

7523AX

7523AZ

7523BA

7523BB

7523BC

7523BD

7523BE

7523BG

7523BH

7523BJ

7523BK

7523BL

7523BM

7523BP

7523BR

7523BS

7523BT

7523BV

7523CB

7523CC

7523CD

7523DB

7523DC

7523DD

7523DE

7523DG

7523DH

7523DK

7523DL

7523DM

7523DN

7523DP

7523DR

7523DS

7523EA

7523EB

7523EC

7523EG

7523EH

7523EN

7523JD

7523JE

7523JG

7523JH

7523JJ

7523JK

7523JL

7523JM

7523MA

7523MB

7523MC

7523MD

7523WB

7523WG

7523WN

7523WZ

7523XA

7523XB

7523XC

7523XD

7523XE

7523XG

7523XH

7523XJ

7523XK

7523XL

7523XM

7523XN

7523XP

7523XR

7523XS

7523XT

7523XV

7523XW

7523XX

7523XZ

7523ZA

7523ZB

7523ZC

7523ZD

7523ZE

7523ZG

7523ZH

7523ZK

7523ZL

7523ZM

7523ZN

7523ZP

7523ZR

7523ZS

7523ZT

7523ZV

7523ZW

7523ZX
