# Supplementary material for: Wages and employment security following a major disaster: A 17-year population-based longitudinal comparative study
Source: PLoS One. 2019 Mar 29;14(3):e0214208. doi: 10.1371/journal.pone.0214208 (PMC6440641; doi:10.1371/journal.pone.0214208)
Supplement: S2 Appendix — (DOCX) [file pone.0214208.s002.docx]

**S2 Appendix Annual gross wages affected and non-affected control groups**

|  |  | Predicted | | |  | Observed | | |
| --- | --- | --- | --- | --- | --- | --- | --- | --- |
|  |  | Non-affected residents Netherlands | Affected residents Enschede inner area | Non-affected residents Tilburg |  | Non-affected residents Netherlands | Affected residents Enschede inner area | Non-affected residents Tilburg |
| 1999 | Mean | 11303.54 | 10450.45 | 10326.79 |  | 10106.88 | 9774.56 | 10004.07 |
|  | SD | 5380.766 | 5192.185 | 5170.847 |  | 13015.217 | 12100.7 | 12841.88 |
|  | N | 3028 | 3044 | 3038 |  | 3028 | 3044 | 3038 |
|  |  |  |  |  |  |  |  |  |
| 2000 | Mean | 12409.69 | 11353.27 | 11441.22 |  | 11284.20 | 10725.74 | 11222.49 |
|  | SD | 5528.74 | 5323.18 | 5314.57 |  | 13832.748 | 12660.32 | 13827.64 |
|  | N | 3054 | 3064 | 3052 |  | 3054 | 3064 | 3052 |
|  |  |  |  |  |  |  |  |  |
| 2001 | Mean | 13558.09 | 12282.58 | 12444.85 |  | 12612.61 | 11836.09 | 12384.06 |
|  | SD | 5774.67 | 5628.40 | 5624.69 |  | 15293.364 | 14301.95 | 14518.75 |
|  | N | 3044 | 3049 | 3055 |  | 3044 | 3049 | 3055 |
|  |  |  |  |  |  |  |  |  |
| 2002 | Mean | 14432.69 | 12997.66 | 13297.57 |  | 13659.63 | 12671.43 | 13339.01 |
|  | SD | 5929.89 | 5763.75 | 5833.68 |  | 16472.929 | 13847.16 | 15165.61 |
|  | N | 3016 | 3040 | 3033 |  | 3016 | 3040 | 3033 |
|  |  |  |  |  |  |  |  |  |
| 2003 | Mean | 15069.38 | 13638.53 | 13843.87 |  | 14463.90 | 13427.39 | 14098.46 |
|  | SD | 6127.38 | 5967.10 | 6148.84 |  | 17366.596 | 14804.84 | 16023.85 |
|  | N | 2990 | 3026 | 3000 |  | 2990 | 3026 | 3000 |
|  |  |  |  |  |  |  |  |  |
| 2004 | Mean | 15360.60 | 13896.23 | 14300.50 |  | 14828.33 | 13777.62 | 14751.61 |
|  | SD | 6317.81 | 6134.21 | 6368.84 |  | 17830.354 | 15772.49 | 18026.18 |
|  | N | 2976 | 3011 | 2978 |  | 2976 | 3011 | 2978 |
|  |  |  |  |  |  |  |  |  |
| 2005 | Mean | 15850.01 | 14048.82 | 14707.16 |  | 15453.02 | 14021.47 | 15335.95 |
|  | SD | 6535.45 | 6371.53 | 6633.36 |  | 18537.119 | 15262.74 | 19411.44 |
|  | N | 2959 | 2988 | 2947 |  | 2959 | 2988 | 2947 |
|  |  |  |  |  |  |  |  |  |
| 2006 | Mean | 17160.78 | 15423.46 | 15725.75 |  | 16792.95 | 15463.05 | 16495.03 |
|  | SD | 7096.56 | 6976.91 | 7250.20 |  | 20468.681 | 17175.8 | 19730.25 |
|  | N | 2951 | 2980 | 2916 |  | 2951 | 2980 | 2916 |
|  |  |  |  |  |  |  |  |  |
| 2007 | Mean | 18396.80 | 16650.95 | 16874.05 |  | 18095.92 | 16717.33 | 17655.03 |
|  | SD | 7237.98 | 7134.24 | 7373.52 |  | 21103.948 | 18121.67 | 19948.09 |
|  | N | 2950 | 2967 | 2902 |  | 2950 | 2967 | 2902 |
|  |  |  |  |  |  |  |  |  |
| 2008 | Mean | 19765.63 | 18031.76 | 18189.38 |  | 19473.38 | 18176.19 | 19046.42 |
|  | SD | 7384.60 | 7356.07 | 7564.68 |  | 24603.957 | 19447.68 | 21181.65 |
|  | N | 2937 | 2945 | 2883 |  | 2937 | 2945 | 2883 |
|  |  |  |  |  |  |  |  |  |
| 2009 | Mean | 20436.50 | 18602.64 | 18342.67 |  | 20206.73 | 18712.55 | 19218.06 |
|  | SD | 7614.13 | 7528.72 | 7795.75 |  | 23290.584 | 20561.26 | 21774.01 |
|  | N | 2919 | 2949 | 2882 |  | 2919 | 2949 | 2882 |
|  |  |  |  |  |  |  |  |  |
| 2010 | Mean | 20363.98 | 18790.24 | 18708.70 |  | 20160.60 | 18948.33 | 19615.66 |
|  | SD | 7751.41 | 7677.75 | 7883.42 |  | 23267.284 | 20795.73 | 22874.84 |
|  | N | 2895 | 2924 | 2882 |  | 2895 | 2924 | 2882 |
|  |  |  |  |  |  |  |  |  |
| 2011 | Mean | 21433.11 | 19378.99 | 19542.63 |  | 21260.52 | 19543.39 | 20491.53 |
|  | SD | 7846.43 | 7858.04 | 8039.42 |  | 24533.867 | 21665.67 | 24143.06 |
|  | N | 2877 | 2917 | 2854 |  | 2877 | 2917 | 2854 |
|  |  |  |  |  |  |  |  |  |
| 2012 | Mean | 21604.87 | 19825.17 | 19880.56 |  | 21349.31 | 19959.68 | 20785.56 |
|  | SD | 7929.03 | 8033.82 | 8155.11 |  | 24873.148 | 22607.01 | 24382.93 |
|  | N | 2873 | 2898 | 2841 |  | 2873 | 2898 | 2841 |
|  |  |  |  |  |  |  |  |  |
| 2013 | Mean | 20891.73 | 18759.29 | 18916.24 |  | 20577.77 | 18910.54 | 19843.72 |
|  | SD | 7979.58 | 8155.88 | 8371.64 |  | 28634.258 | 22252.98 | 24333.65 |
|  | N | 2851 | 2883 | 2808 |  | 2851 | 2883 | 2808 |
|  |  |  |  |  |  |  |  |  |
| 2014 | Mean | 21399.16 | 19129.32 | 19062.59 |  | 21035.10 | 19262.69 | 19995.12 |
|  | SD | 8097.97 | 8208.12 | 8446.13 |  | 28915.901 | 23117.79 | 24334.39 |
|  | N | 2833 | 2879 | 2779 |  | 2833 | 2879 | 2779 |
|  |  |  |  |  |  |  |  |  |
| 2015 | Mean | 22047.68 | 19836.48 | 19805.99 |  | 21642.93 | 19961.86 | 20727.56 |
|  | SD | 8395.18 | 8541.10 | 8828.82 |  | 27576.492 | 24471.96 | 25604.91 |
|  | N | 2807 | 2850 | 2753 |  | 2807 | 2850 | 2753 |
|  |  |  |  |  |  |  |  |  |
| 2016 | Mean | 22935.98 | 20796.86 | 20898.86 |  | 22533.73 | 20921.67 | 21797.74 |
|  | SD | 8490.39 | 8623.61 | 8921.05 |  | 28626.659 | 25189.2 | 25811.08 |
|  | N | 2778 | 2808 | 2722 |  | 2778 | 2808 | 2722 |
|  |  |  |  |  |  |  |  |  |
