# Supplementary material for: Wages and employment security following a major disaster: A 17-year population-based longitudinal comparative study
Source: PLoS One. 2019 Mar 29;14(3):e0214208. doi: 10.1371/journal.pone.0214208 (PMC6440641; doi:10.1371/journal.pone.0214208)
Supplement: S3 Appendix — (DOCX) [file pone.0214208.s003.docx]

**S3 Appendix Annual gross wages affected residents and control groups with low wages (low-wage groups) in 1999**

|  |  | Predicted | | |  |  | Observed | | |
| --- | --- | --- | --- | --- | --- | --- | --- | --- | --- |
|  |  | Non-affected residents Netherlands | Affected residents Enschede inner area | Non-affected residents Tilburg |  |  | Non-affected residents Netherlands | Affected residents Enschede inner area | Non-affected residents Tilburg |
| 1999 | Mean | 2560.06 | 1723.04 | 1787.72 |  |  | 1811.77 | 1739.91 | 1780.52 |
|  | SD | 4490.863 | 4166.294 | 4209.611 |  |  | 1166.43 | 1115.93 | 1149.41 |
|  | N | 535 | 533 | 530 |  |  | 535 | 533 | 530 |
|  |  |  |  |  |  |  |  |  |  |
| 2000 | Mean | 4493.80 | 3379.84 | 3843.10 |  |  | 3764.34 | 3421.65 | 3744.34 |
|  | SD | 5521.71 | 5486.53 | 4965.09 |  |  | 4403.31 | 4144.21 | 4322.79 |
|  | N | 544 | 544 | 544 |  |  | 544 | 544 | 544 |
|  |  |  |  |  |  |  |  |  |  |
| 2001 | Mean | 7172.98 | 5628.77 | 6511.00 |  |  | 6468.46 | 5626.03 | 6476.37 |
|  | SD | 6413.25 | 6368.67 | 6193.43 |  |  | 7094.14 | 6825.47 | 7097.58 |
|  | N | 546 | 546 | 540 |  |  | 546 | 546 | 545 |
|  |  |  |  |  |  |  |  |  |  |
| 2002 | Mean | 9415.59 | 8276.15 | 8805.94 |  |  | 8719.11 | 8285.55 | 8741.42 |
|  | SD | 6928.16 | 6750.67 | 6874.91 |  |  | 9217.39 | 8906.76 | 9024.40 |
|  | N | 544 | 545 | 538 |  |  | 544 | 545 | 543 |
|  |  |  |  |  |  |  |  |  |  |
| 2003 | Mean | 11499.04 | 10422.65 | 10936.75 |  |  | 10840.62 | 10455.75 | 10896.45 |
|  | SD | 7568.48 | 7084.76 | 7587.88 |  |  | 10688.63 | 10339.07 | 10612.89 |
|  | N | 539 | 543 | 533 |  |  | 539 | 543 | 538 |
|  |  |  |  |  |  |  |  |  |  |
| 2004 | Mean | 12800.44 | 12156.97 | 12869.08 |  |  | 12183.43 | 12221.22 | 12849.07 |
|  | SD | 7837.29 | 7525.19 | 7862.21 |  |  | 11760.36 | 11733.11 | 11899.25 |
|  | N | 535 | 541 | 528 |  |  | 535 | 541 | 532 |
|  |  |  |  |  |  |  |  |  |  |
| 2005 | Mean | 14407.12 | 14226.28 | 14613.04 |  |  | 13838.32 | 14300.60 | 14699.57 |
|  | SD | 8263.16 | 7974.60 | 8345.47 |  |  | 12595.28 | 12981.31 | 13327.65 |
|  | N | 529 | 535 | 521 |  |  | 529 | 535 | 524 |
|  |  |  |  |  |  |  |  |  |  |
| 2006 | Mean | 17564.90 | 16955.28 | 16974.77 |  |  | 17008.89 | 17060.60 | 17062.72 |
|  | SD | 9012.87 | 8841.66 | 9441.34 |  |  | 14812.60 | 14989.48 | 15039.23 |
|  | N | 524 | 532 | 519 |  |  | 524 | 532 | 522 |
|  |  |  |  |  |  |  |  |  |  |
| 2007 | Mean | 20543.79 | 19778.16 | 19581.17 |  |  | 19973.58 | 19922.58 | 19703.87 |
|  | SD | 9222.83 | 9148.42 | 9601.62 |  |  | 16311.28 | 17192.75 | 17291.45 |
|  | N | 522 | 528 | 515 |  |  | 522 | 528 | 518 |
|  |  |  |  |  |  |  |  |  |  |
| 2008 | Mean | 23507.29 | 22069.81 | 21887.14 |  |  | 22939.54 | 22216.45 | 22032.69 |
|  | SD | 9499.83 | 9602.02 | 9792.21 |  |  | 18806.82 | 19158.25 | 18499.14 |
|  | N | 518 | 524 | 508 |  |  | 518 | 524 | 511 |
|  |  |  |  |  |  |  |  |  |  |
| 2009 | Mean | 24893.70 | 23379.92 | 22920.14 |  |  | 24396.34 | 23584.44 | 23083.17 |
|  | SD | 10051.03 | 9851.70 | 10151.65 |  |  | 21034.23 | 21143.74 | 19419.58 |
|  | N | 510 | 520 | 504 |  |  | 510 | 520 | 507 |
|  |  |  |  |  |  |  |  |  |  |
| 2010 | Mean | 26107.67 | 23942.08 | 24044.97 |  |  | 25704.15 | 24173.84 | 24301.32 |
|  | SD | 10255.55 | 10183.37 | 10401.13 |  |  | 21988.16 | 22197.21 | 21232.20 |
|  | N | 504 | 515 | 500 |  |  | 504 | 515 | 503 |
|  |  |  |  |  |  |  |  |  |  |
| 2011 | Mean | 28098.99 | 25602.65 | 26410.79 |  |  | 27788.80 | 25872.77 | 26772.45 |
|  | SD | 10456.65 | 10577.10 | 10583.22 |  |  | 23777.23 | 24694.58 | 23249.77 |
|  | N | 499 | 512 | 493 |  |  | 499 | 512 | 496 |
|  |  |  |  |  |  |  |  |  |  |
| 2012 | Mean | 29040.30 | 26523.76 | 27139.90 |  |  | 28830.97 | 26836.42 | 27487.36 |
|  | SD | 10543.49 | 11093.47 | 10769.84 |  |  | 25316.27 | 26418.69 | 24426.59 |
|  | N | 491 | 506 | 487 |  |  | 491 | 506 | 490 |
|  |  |  |  |  |  |  |  |  |  |
| 2013 | Mean | 27898.75 | 25901.48 | 26357.95 |  |  | 27673.48 | 26292.95 | 26843.00 |
|  | SD | 10677.93 | 11283.15 | 11280.66 |  |  | 27693.57 | 27434.41 | 24802.43 |
|  | N | 489 | 500 | 478 |  |  | 489 | 500 | 481 |
|  |  |  |  |  |  |  |  |  |  |
| 2014 | Mean | 28558.60 | 26700.03 | 27396.86 |  |  | 28450.78 | 27197.71 | 27999.43 |
|  | SD | 10917.43 | 11371.40 | 11415.29 |  |  | 29062.14 | 28883.37 | 26096.58 |
|  | N | 479 | 492 | 471 |  |  | 479 | 492 | 474 |
|  |  |  |  |  |  |  |  |  |  |
| 2015 | Mean | 30286.25 | 28633.62 | 28419.83 |  |  | 30269.69 | 29305.39 | 29084.90 |
|  | SD | 11149.59 | 11787.77 | 11889.33 |  |  | 32275.85 | 31063.25 | 28836.59 |
|  | N | 473 | 482 | 464 |  |  | 473 | 482 | 467 |
|  |  |  |  |  |  |  |  |  |  |
| 2016 | Mean | 31088.10 | 30230.34 | 29813.54 |  |  | 31030.93 | 31096.58 | 30517.30 |
|  | SD | 11154.22 | 11905.97 | 11879.09 |  |  | 34905.32 | 32621.41 | 28883.84 |
|  | N | 467 | 474 | 462 |  |  | 467 | 474 | 465 |
|  |  |  |  |  |  |  |  |  |  |
