# Supplementary material for: Wages and employment security following a major disaster: A 17-year population-based longitudinal comparative study
Source: PLoS One. 2019 Mar 29;14(3):e0214208. doi: 10.1371/journal.pone.0214208 (PMC6440641; doi:10.1371/journal.pone.0214208)
Supplement: S4 Appendix — (DOCX) [file pone.0214208.s004.docx]

**S4 Appendix Employment security affected and non-affected control groups**

|  |  | Predicted | | |  | Observed | | |
| --- | --- | --- | --- | --- | --- | --- | --- | --- |
|  |  | Non-affected residents Netherlands | Affected residents Enschede inner area | Non-affected residents Tilburg |  | Non-affected residents Netherlands | Affected residents Enschede inner area | Non-affected residents Tilburg |
| 1999 | Mean | 32.31 | 32.53 | 32.74 |  | 28.86 | 28.81 | 28.85 |
|  | SD | 4.07 | 4.19 | 4.01 |  | 24.00 | 24.03 | 23.95 |
|  | N | 3028 | 3044 | 3038 |  | 3028 | 3044 | 3038 |
|  |  |  |  |  |  |  |  |  |
| 2000 | Mean | 32.77 | 33.23 | 33.54 |  | 29.62 | 29.79 | 30.10 |
|  | SD | 4.11 | 4.15 | 4.07 |  | 24.12 | 23.99 | 23.89 |
|  | N | 3054 | 3064 | 3052 |  | 3054 | 3064 | 3052 |
|  |  |  |  |  |  |  |  |  |
| 2001 | Mean | 33.03 | 33.08 | 33.77 |  | 30.40 | 30.29 | 30.87 |
|  | SD | 4.32 | 4.36 | 4.33 |  | 24.05 | 23.95 | 23.77 |
|  | N | 3044 | 3049 | 3055 |  | 3044 | 3049 | 3055 |
|  |  |  |  |  |  |  |  |  |
| 2002 | Mean | 32.73 | 33.35 | 34.02 |  | 30.67 | 31.08 | 31.59 |
|  | SD | 4.55 | 4.44 | 4.52 |  | 24.08 | 24.03 | 23.75 |
|  | N | 3016 | 3040 | 3033 |  | 3016 | 3040 | 3033 |
|  |  |  |  |  |  |  |  |  |
| 2003 | Mean | 32.53 | 33.00 | 33.45 |  | 31.06 | 31.20 | 31.71 |
|  | SD | 4.75 | 4.70 | 4.78 |  | 24.24 | 24.04 | 23.93 |
|  | N | 2990 | 3026 | 3000 |  | 2990 | 3026 | 3000 |
|  |  |  |  |  |  |  |  |  |
| 2004 | Mean | 31.83 | 32.23 | 32.41 |  | 30.71 | 30.78 | 31.35 |
|  | SD | 4.97 | 4.87 | 5.02 |  | 24.34 | 24.24 | 23.97 |
|  | N | 2976 | 3011 | 2978 |  | 2976 | 3011 | 2978 |
|  |  |  |  |  |  |  |  |  |
| 2005 | Mean | 31.63 | 31.89 | 31.95 |  | 31.04 | 30.79 | 31.45 |
|  | SD | 5.12 | 4.99 | 5.14 |  | 24.34 | 24.19 | 24.06 |
|  | N | 2959 | 2988 | 2947 |  | 2959 | 2988 | 2947 |
|  |  |  |  |  |  |  |  |  |
| 2006 | Mean | 31.50 | 32.02 | 32.20 |  | 31.26 | 31.40 | 32.22 |
|  | SD | 5.40 | 5.31 | 5.51 |  | 23.91 | 23.92 | 23.69 |
|  | N | 2951 | 2980 | 2916 |  | 2951 | 2980 | 2916 |
|  |  |  |  |  |  |  |  |  |
| 2007 | Mean | 31.82 | 32.73 | 32.59 |  | 32.06 | 32.51 | 33.08 |
|  | SD | 5.53 | 5.45 | 5.58 |  | 23.86 | 23.74 | 23.44 |
|  | N | 2950 | 2967 | 2902 |  | 2950 | 2967 | 2902 |
|  |  |  |  |  |  |  |  |  |
| 2008 | Mean | 31.99 | 32.98 | 32.80 |  | 32.62 | 33.21 | 33.62 |
|  | SD | 5.56 | 5.57 | 5.62 |  | 23.71 | 23.53 | 23.40 |
|  | N | 2937 | 2945 | 2883 |  | 2937 | 2945 | 2883 |
|  |  |  |  |  |  |  |  |  |
| 2009 | Mean | 31.37 | 31.87 | 31.62 |  | 32.45 | 32.38 | 32.76 |
|  | SD | 5.66 | 5.63 | 5.67 |  | 23.81 | 23.85 | 23.59 |
|  | N | 2919 | 2949 | 2882 |  | 2919 | 2949 | 2882 |
|  |  |  |  |  |  |  |  |  |
| 2010 | Mean | 30.70 | 31.82 | 31.02 |  | 32.28 | 32.78 | 32.49 |
|  | SD | 5.78 | 5.75 | 5.73 |  | 23.79 | 23.75 | 23.68 |
|  | N | 2895 | 2924 | 2882 |  | 2895 | 2924 | 2882 |
|  |  |  |  |  |  |  |  |  |
| 2011 | Mean | 30.96 | 31.64 | 31.11 |  | 32.80 | 32.95 | 32.91 |
|  | SD | 5.86 | 5.86 | 5.90 |  | 23.72 | 23.61 | 23.65 |
|  | N | 2877 | 2917 | 2854 |  | 2877 | 2917 | 2854 |
|  |  |  |  |  |  |  |  |  |
| 2012 | Mean | 30.27 | 31.09 | 30.65 |  | 32.37 | 32.61 | 32.69 |
|  | SD | 5.94 | 5.94 | 5.91 |  | 23.82 | 23.79 | 23.73 |
|  | N | 2873 | 2898 | 2841 |  | 2873 | 2898 | 2841 |
|  |  |  |  |  |  |  |  |  |
| 2013 | Mean | 29.35 | 30.17 | 29.68 |  | 31.81 | 31.99 | 32.10 |
|  | SD | 5.94 | 5.99 | 5.99 |  | 23.91 | 23.95 | 23.87 |
|  | N | 2851 | 2883 | 2808 |  | 2851 | 2883 | 2808 |
|  |  |  |  |  |  |  |  |  |
| 2014 | Mean | 29.12 | 29.72 | 29.20 |  | 31.85 | 31.82 | 31.90 |
|  | SD | 6.06 | 5.97 | 6.12 |  | 23.99 | 24.01 | 24.10 |
|  | N | 2833 | 2879 | 2779 |  | 2833 | 2879 | 2779 |
|  |  |  |  |  |  |  |  |  |
| 2015 | Mean | 28.99 | 29.58 | 28.95 |  | 32.04 | 31.99 | 31.89 |
|  | SD | 6.25 | 6.28 | 6.40 |  | 23.90 | 23.80 | 23.98 |
|  | N | 2807 | 2850 | 2753 |  | 2807 | 2850 | 2753 |
|  |  |  |  |  |  |  |  |  |
| 2016 | Mean | 29.32 | 30.05 | 29.79 |  | 32.56 | 32.62 | 32.93 |
|  | SD | 6.38 | 6.43 | 6.46 |  | 23.86 | 23.66 | 23.70 |
|  | N | 2778 | 2808 | 2722 |  | 2778 | 2808 | 2722 |
|  |  |  |  |  |  |  |  |  |
