# Supplementary material for: Wages and employment security following a major disaster: A 17-year population-based longitudinal comparative study
Source: PLoS One. 2019 Mar 29;14(3):e0214208. doi: 10.1371/journal.pone.0214208 (PMC6440641; doi:10.1371/journal.pone.0214208)
Supplement: S5 Appendix — (DOCX) [file pone.0214208.s005.docx]

**S5 Appendix Employment security affected residents and non-affected controls groups with low wages in 1999 (low-wage group)**

|  |  | Predicted | | |  | Observed | | |
| --- | --- | --- | --- | --- | --- | --- | --- | --- |
|  |  | Non-affected residents Netherlands | Affected residents Enschede inner area | Non-affected residents Tilburg |  | Non-affected residents Netherlands | Affected residents Enschede inner area | Non-affected residents Tilburg |
| 1999 | Mean | 28.61 | 28.26 | 28.28 |  | 27.44 | 26.95 | 28.25 |
|  | SD | 2.667 | 2.645 | 2.665 |  | 18.43 | 18.16 | 18.29 |
|  | N | 535 | 533 | 530 |  | 535 | 533 | 530 |
|  |  |  |  |  |  |  |  |  |
| 2000 | Mean | 30.97 | 30.25 | 31.38 |  | 30.06 | 29.19 | 31.26 |
|  | SD | 3.73 | 3.98 | 3.42 |  | 20.85 | 20.66 | 20.49 |
|  | N | 544 | 544 | 544 |  | 544 | 544 | 544 |
|  |  |  |  |  |  |  |  |  |
| 2001 | Mean | 33.71 | 31.15 | 34.24 |  | 32.78 | 30.14 | 34.34 |
|  | SD | 4.50 | 4.80 | 4.29 |  | 20.89 | 21.62 | 20.45 |
|  | N | 546 | 546 | 540 |  | 546 | 546 | 545 |
|  |  |  |  |  |  |  |  |  |
| 2002 | Mean | 35.17 | 33.92 | 36.13 |  | 34.35 | 32.95 | 36.12 |
|  | SD | 5.09 | 5.23 | 4.95 |  | 21.00 | 21.64 | 20.44 |
|  | N | 544 | 545 | 538 |  | 544 | 545 | 543 |
|  |  |  |  |  |  |  |  |  |
| 2003 | Mean | 36.56 | 35.74 | 36.28 |  | 35.83 | 34.87 | 36.40 |
|  | SD | 5.56 | 5.52 | 5.53 |  | 21.11 | 21.62 | 21.13 |
|  | N | 539 | 543 | 533 |  | 539 | 543 | 538 |
|  |  |  |  |  |  |  |  |  |
| 2004 | Mean | 36.12 | 35.52 | 36.02 |  | 35.42 | 34.65 | 36.24 |
|  | SD | 5.83 | 5.76 | 5.78 |  | 21.75 | 22.13 | 21.09 |
|  | N | 535 | 541 | 528 |  | 535 | 541 | 532 |
|  |  |  |  |  |  |  |  |  |
| 2005 | Mean | 36.26 | 36.74 | 36.39 |  | 35.75 | 35.96 | 36.94 |
|  | SD | 5.95 | 6.07 | 6.01 |  | 21.77 | 21.73 | 21.33 |
|  | N | 529 | 535 | 521 |  | 529 | 535 | 524 |
|  |  |  |  |  |  |  |  |  |
| 2006 | Mean | 37.37 | 37.60 | 37.07 |  | 36.98 | 36.90 | 37.63 |
|  | SD | 6.13 | 6.22 | 6.50 |  | 21.25 | 21.54 | 21.21 |
|  | N | 524 | 532 | 519 |  | 524 | 532 | 522 |
|  |  |  |  |  |  |  |  |  |
| 2007 | Mean | 39.33 | 38.76 | 37.25 |  | 38.95 | 38.16 | 37.98 |
|  | SD | 6.23 | 6.31 | 6.52 |  | 20.60 | 21.28 | 21.16 |
|  | N | 522 | 528 | 515 |  | 522 | 528 | 518 |
|  |  |  |  |  |  |  |  |  |
| 2008 | Mean | 39.62 | 38.94 | 37.58 |  | 39.42 | 38.40 | 38.35 |
|  | SD | 6.23 | 6.49 | 6.55 |  | 20.48 | 21.16 | 21.07 |
|  | N | 518 | 524 | 508 |  | 518 | 524 | 511 |
|  |  |  |  |  |  |  |  |  |
| 2009 | Mean | 37.89 | 37.76 | 37.07 |  | 37.85 | 37.32 | 37.95 |
|  | SD | 6.31 | 6.52 | 6.57 |  | 21.52 | 21.99 | 21.57 |
|  | N | 510 | 520 | 504 |  | 510 | 520 | 507 |
|  |  |  |  |  |  |  |  |  |
| 2010 | Mean | 37.99 | 37.16 | 36.39 |  | 38.15 | 36.71 | 37.43 |
|  | SD | 6.37 | 6.50 | 6.58 |  | 21.31 | 22.29 | 22.03 |
|  | N | 504 | 515 | 500 |  | 504 | 515 | 503 |
|  |  |  |  |  |  |  |  |  |
| 2011 | Mean | 38.58 | 36.99 | 37.07 |  | 38.91 | 36.59 | 38.22 |
|  | SD | 6.41 | 6.57 | 6.62 |  | 21.18 | 22.57 | 21.47 |
|  | N | 499 | 512 | 493 |  | 499 | 512 | 496 |
|  |  |  |  |  |  |  |  |  |
| 2012 | Mean | 38.35 | 36.40 | 36.72 |  | 38.74 | 36.07 | 37.96 |
|  | SD | 6.43 | 6.66 | 6.52 |  | 21.27 | 22.99 | 21.61 |
|  | N | 491 | 506 | 487 |  | 491 | 506 | 490 |
|  |  |  |  |  |  |  |  |  |
| 2013 | Mean | 36.67 | 35.79 | 36.26 |  | 37.12 | 35.49 | 37.64 |
|  | SD | 6.40 | 6.65 | 6.59 |  | 22.11 | 23.37 | 22.08 |
|  | N | 489 | 500 | 478 |  | 489 | 500 | 481 |
|  |  |  |  |  |  |  |  |  |
| 2014 | Mean | 36.33 | 35.21 | 35.99 |  | 36.98 | 35.14 | 37.62 |
|  | SD | 6.37 | 6.64 | 6.55 |  | 22.54 | 23.45 | 22.23 |
|  | N | 479 | 492 | 471 |  | 479 | 492 | 474 |
|  |  |  |  |  |  |  |  |  |
| 2015 | Mean | 35.59 | 35.54 | 34.79 |  | 36.33 | 35.73 | 36.59 |
|  | SD | 6.44 | 6.69 | 6.67 |  | 22.80 | 23.24 | 22.69 |
|  | N | 473 | 482 | 464 |  | 473 | 482 | 467 |
|  |  |  |  |  |  |  |  |  |
| 2016 | Mean | 35.11 | 35.55 | 35.76 |  | 35.85 | 35.94 | 37.63 |
|  | SD | 6.38 | 6.70 | 6.62 |  | 23.12 | 22.94 | 22.42 |
|  | N | 467 | 474 | 462 |  | 467 | 474 | 465 |
|  |  |  |  |  |  |  |  |  |
